# The prognostic value of the visually assessed time difference between mitral valve and tricuspid valve opening score for patients with heart failure with mildly reduced ejection fraction

**DOI:** 10.1002/clc.24223

**Published:** 2024-01-29

**Authors:** Yin Yue, Xiaopeng Wu, Xiaonan Guan, Xuejiao Wu, Jianjun Zhang

**Affiliations:** ^1^ Department of Cardiology, Beijing Chaoyang Hospital Capital Medical University Beijing China

**Keywords:** cardiovascular disease‐cause mortality, heart failure with mildly reduced ejection fraction, the VMT score

## Abstract

**Background:**

The visually assessed time difference between the mitral valve and tricuspid valve opening (VMT) score was correlated with the increase of left ventricular filling pressure (LVFP).

**Hypothesis:**

We suspected that the VMT score might be a valuable prognostic biomarker for heart failure with mildly reduced ejection fraction (HFmrEF) patients. This study was to evaluate the predictive value of VMT score for 1‐year all‐cause and cardiovascular disease (CVD)‐cause mortality in HFmrEF patients.

**Methods:**

This cohort study enrolled 379 patients aged ≥18 years old with HFmrEF. Univariable and multivariable Cox regression analysis was employed to assess the association between VMT score and all‐cause or CVD‐cause mortality in HFmrEF patients. Hazards ratio (HR), and 95% confidence interval (CI) were effect sizes. Kaplan–Meier curves showed the survival probability of patients. The area under the curve (AUC) evaluated the prognostic value of the VMT score.

**Results:**

The risk of all‐cause mortality was increased in HFmrEF patients in the VMT score of 2 (HR = 2.80, 95%CI: 1.04–7.52) and 3 (HR = 4.29, 95%CI: 1.58–11.66). The VMT score of 3 was associated with an increased risk of 1‐year CVD‐cause mortality in patients with HFmrEF (HR = 7.63, 95%CI: 1.70–34.33). The AUC of VMT score for predicting 1‐year all‐cause mortality of HFmrEF patients was 0.724, and for predicting 1‐year CVD‐cause mortality of HFmrEF patients was 0.748. The survival probability of patients with the VMT score < 2 was higher than those with the VMT score of 2 and 3.

**Conclusion:**

The VMT score might be a reliable prognostic index for 1‐year all‐cause or CVD‐cause mortality of HFmrEF patients.

## Graphical abstract

This cohort study evaluated the associations between the mitral valve and tricuspid valve opening (VMT) score and 1‐year all‐cause or cardiovascular disease (CVD)‐cause mortality of heart failure with mildly reduced ejection fraction (HFmrEF) patients using eligible participants' data. The predictive values of the VMT score for the 1‐year all‐cause and CVD‐cause mortality in HFmrEF patients were also evaluated.

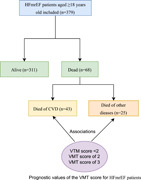

## 1 INTRODUCTION

Heart failure (HF) is a global pandemic with increasing prevalence, and the prevalence of HF was 1.3% in China.^1^, ^2^ HF is the leading cause of hospitalization among adults, and the 1‐year mortality was reported to range from 10% to 35%.^3^ Based on the left ventricular ejection fraction (LVEF), heart failure can be classified into HF with reduced ejection fraction (HFrEF) (LVEF < 40%) and HF with preserved ejection fraction (HFpEF) (LVEF ≥ 50%).^4^ The range between these two fractions is called HF with mildly reduced ejection fraction (HFmrEF), referring to an LVEF of 41%–49%.^5^ HFmrEF is an under‐recognized type of HF, and the prevalence of was 10%–25% in all HF cases.^6^ HFmrEF has some intermediate features between HFrEF and HFpEF, but HFmrEF, HFrEF and HFpEF exhibited heterogeneity in presentation and pathophysiology, which played an essential part on the prognosis and treatment of the disease.^7^, ^8^ Patients with HFmrEF have a lower risk of cardiovascular disease (CVD)‐cause mortality than patients with HFrEF and have similar or greater risk of non‐CVD‐cause mortality than those with HFrEF.^6^ To clearly identify biomarkers associated with the prognosis of patients with HFmrEF was of great value.

Cardiac remodeling is one of the mechanism of HF, and cardiac volume and pressure overload was an important factor in promoting cardiac remodeling, which played an essential role in the occurrence and development of HF.^9^, ^10^ Although HFmrEF was reported to be a heterogeneous group, there may be no single pathophysiological mechanism. Echocardiography is the most valuable examination method for quantitative or qualitative detection of atrioventricular‐related parameters, which provides objective indicators for the evaluation and treatment of HF.^11^ Recently, the visually assessed time difference between the mitral valve (MV) and tricuspid valve (TV) opening (VMT), combined with an indicator of right atrial pressure [inferior vena cava (IVC) dimension], formed the VMT score.^12^ The VMT score was reported to be correlated with the increase of left ventricular filling pressure (LVFP),^12^ and served as a useful indicator for evaluating the prognosis of HFpEF.^13^ Thus, we suspected that the VMT score might be a valuable prognostic biomarker for HFmrEF patients.

This study aimed to explore the association between the VMT score and the outcome of HFmrEF patients and evaluate the predictive values of the VMT score for the 1‐year all‐cause and CVD‐cause mortality in HFmrEF patients.

## 2 METHODS

### 2.1 Study design and population

This cohort study enrolled 418 patients aged ≥ 18 years old with HFmrEF in Beijing Chaoyang Hospital, Capital Medical University. HFmrEF was diagnosed according to the 2021 ESC Guidelines for the diagnosis and treatment of acute and chronic HF.^14^ Participants with previous LVEF ≤ 40%, right cardiac decompensation‐related diseases or primary pulmonary hypertension, primary valvular heart disease, or receiving atrioventricular valve replacement, congenital heart disease, constrictive pericardial disease, receiving mechanical circulation or ventilation support and hemodialysis, tumor, severe infection, and those with low quality of ultrasound image were excluded. Finally, 379 patients were included. This study got approval from the Ethics Committee of Chaoyang Hospital, Capital Medical University (No. 2021‐科−713). All participants provided the patient informed consent.

### 2.2 Potential covariates

Demographic variables [age (years), gender (female or male), height (cm), weight (kg), body mass index (BMI) ( ≤ 23.9 kg/m^2^, 23.9–27.9 kg/m^2^ or >27.9 kg/m^2^), smoking (yes or no), and drinking (yes or no)]; complications (hypertension, diabetes, ischemic heart disease, primary myocardiopathy, hyperlipemia, peripheral vascular disease, and stroke), atrial fibrillation (AFIB) and clinical data [history of HF, New York Heart Association (NYHA) classification (II, III, or IV)], clinical symptoms of HF (hypoperfusion or congestion), laboratory data [C‐reactive protein (CRP) (mg/L), lactic acid (LAC) (mmol/L), hemoglobin (g/L), red blood cell (10^12^/L), white blood cell (10^9^/L), platelet (10^9^/L), neutrophil (10^9^/L), lymphocyte (10^9^/L), monocyte (10^9^/L), eosinophil (10^9^/L), basophil (10^9^/L), hematocrit (%), blood urea nitrogen (BUN) (mmol/L), creatinine (μmol/L), estimated glomerular fifiltration rate (eGFR) (mL/min), potassium (mmol/L), sodium (mmol/L), calcium (mmol/L), creatine kinase muscle and brain isoenzyme (CK‐MB) (U/L), myoglobin (MB) (ng/mL), cardiac troponin I (CTNI) (ng/mL), and amino‐terminal pro‐brain natriuretic peptide (NT‐proBNP) (pg/mL)] were potential covariates analyzed.

Echocardiographic data [mitral regurgitation (MR) (yes or no), tricuspid regurgitation (TR) (yes or no), left ventricular end‐diastolic volume (LVEDV) (mL), left ventricular ejection fraction (LVEF) (%), interventricular septum (IVS) (mm), left ventricular mass index (LVMI) (g/m^2^), stroke volume (SV) (mL), transmitral peak early‐diastolic velocity (E) (cm/s), deceleration time of E (EDT) (ms), tricuspid regurgitation pressure gradient (TRPG) (mmHg), left atrial volume index (LAVI) (mL/m^2^), the ratio of early diastolic mitral inflow to mitral annular tissue velocities (E/e'), diameter of right ventricular base (cm), tricuspid annular plane systolic excursion (TAPSE) (mm), right ventricular fractional area change (RVFAC) (%), IVC respiratory change (%)], medication during hospitalization [intravenous diuretics (yes or no), inotropic agents (yes or no)] medication after discharge [beta‐blocker, angiotensin converting enzyme inhibitor (ACEI), and angiotensin receptor blocker (ARB)] were potential covariates analyzed.

### 2.3 Main variable

**Figure 1:**
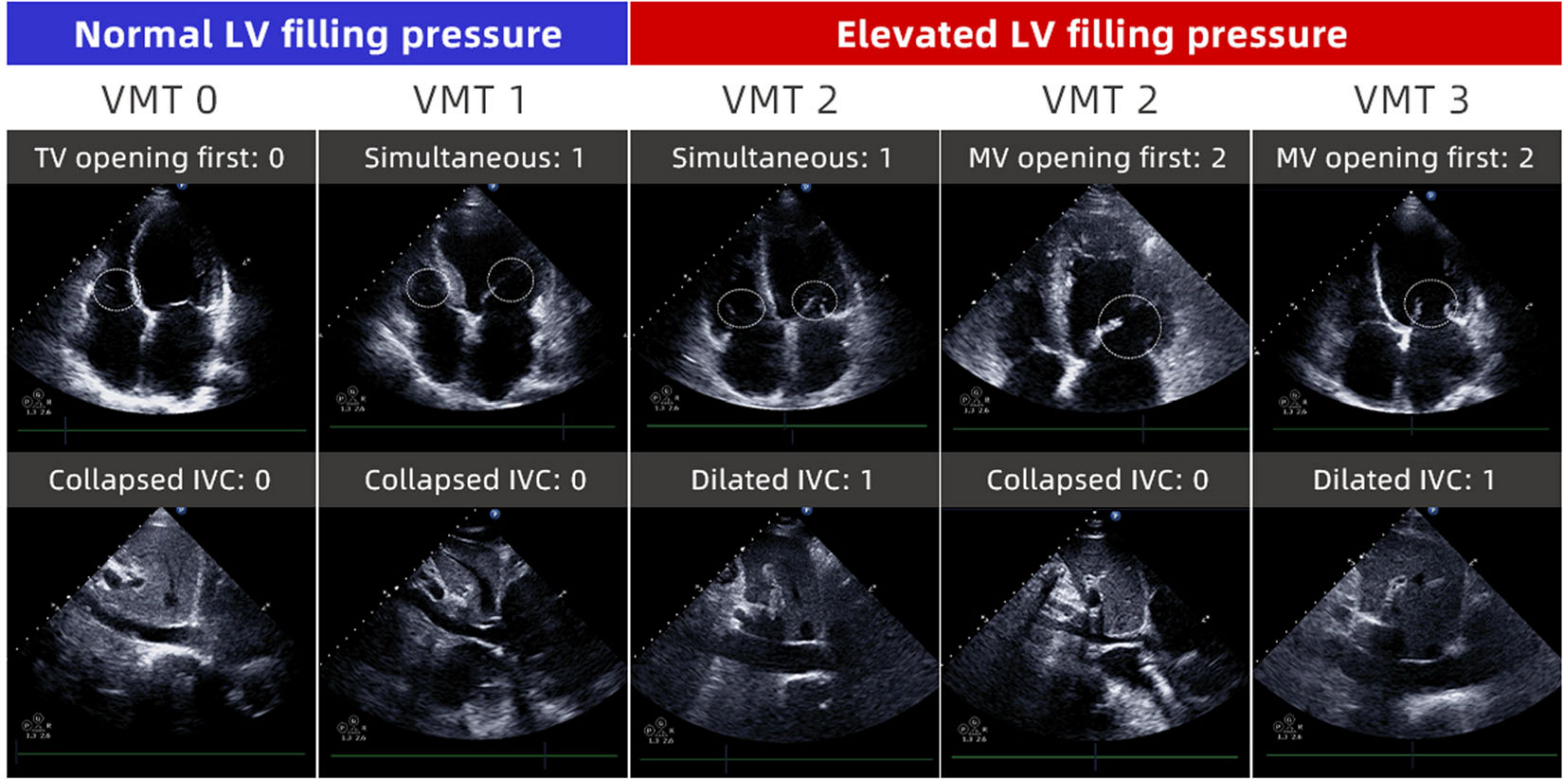
The echocardiographic images of different mitral valve and tricuspid valve opening (VMT) scores.

As the main variable in this study, the VMT score comprises two components: (1) visual assessment of the time sequence of atrioventricular valve openings and (2) estimation of right atrium pressure based on findings from IVC, IVC dimension, and IVC respiratory change. The time sequence of MV and TV openings was visually evaluated according to slow playback from cine loops (6–9 beats) of the apical four‐chamber view. The assessment was categorized into three grades: 0 (TV opening first), 1 (simultaneous opening), and 2 (MV opening first). In cases where markers indicating abnormal RA pressure were observed (IVC dimension >21 mm and IVC respiratory change <50%), an additional point was assigned to calculate the VMT score as a four‐grade scale ranging from 0 to 3^12^ (Figure 1).

### 2.4 Echocardiographic examination

The echocardiographic examination was performed based on the American Society of Echocardiography/European Association of Cardiovascular Imaging guidelines, utilizing Philips CX 50 system (Philips Medical Systems). The examination used a two‐dimensional B mode operating an S5 1‐MHz phased‐array pure‐wave crystal transducer.^15^ The biplane method of disk was employed to measure LVEDV, and LAVI. LVMI was calculated using the Devereux formula. SV was determined by calculating the velocity‐time integral of the LV ejection flow and the cross‐sectional area of the LV outflow tract. Transmitral peak E and EDT were evaluated in the apical LV long‐axis view. The E/e' was the ratio of E to e'. The e' of the two sites at the ventricular septum side and the left ventricular side wall were detected, respectively; early‐diastolic mitral annular velocities at the septal annulus and left ventricular side wall (e') was evaluated from the apical four‐chamber view to calculate the average e'. The IVC dimension and its respiratory change were assessed just proximal to the junction of the hepatic veins.

### 2.5 Outcome variables

The outcomes were 1‐year all‐cause mortality and 1‐year CVD‐cause mortality of patients with HFmrEF. The follow‐up was conducted by telephone at intervals of 1‐, 3‐, 6‐, 9‐, and 12‐month. The median follow‐up time was 12 (12, 12) months.

### 2.6 Statistical analysis

Skewness and kurtosis methods were used to test the normality of measurement data, and Levene test was used to test the homogeneity of variance. Normally distributed measurement data were expressed as mean ± standard deviation (SD). Analysis of variance (ANOVA) was used for comparison among groups with homogeneity of variance, and Kruskal–Wallis H test was used for variance heterogeneity. Non‐normally distributed measurement data were shown as median and quartiles [M (Q_1_, Q_3_)], and Kruskal–Wallis H test was applied for comparison among groups. Enumeration data were exhibited as numbers and percentages of cases [*n* (%)], and *χ* ^2^ test or Fisher's exact test was used for comparison among groups, and Kruskal–Wallis H test was used for comparisons of rank data. The potential confounding factors correlated with all‐cause and CVD‐cause mortality in HFmrEF patients were evaluated via univariate Cox regression analysis. Variables with *p* < .05 were included in the stepwise regression. Univariable and multivariable Cox regression analysis was employed to assess the association between the VMT score and all‐cause or CVD‐cause mortality in HFmrEF patients. Hazards ratio (HR) and 95% confidence interval (CI) were effect sizes. Kaplan–Meier curves were drawn to show the survival probability of patients. Receiver operator characteristic (ROC) curves were plotted, and area under the curve (AUC) was used to evaluate the prognostic value of the VMT score for all‐cause or CVD‐cause mortality in HFmrEF patients. The confidence level was set as *α* = .05. R (version 4.2.3; Institute for Statistics and Mathematics) was utilized for statistical analysis.

## 3 RESULTS

### 3.1 Comparisons of characteristics in HFmrEF patients with and without all‐cause mortality as well as CVD‐cause mortality

**Figure 2:**
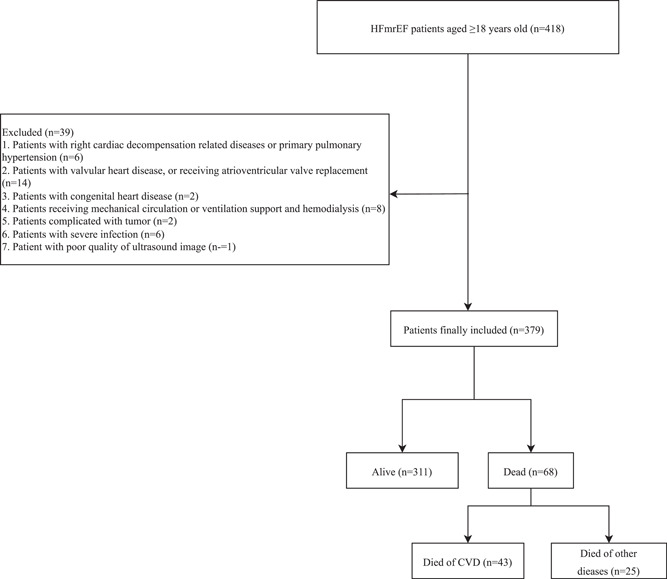
The screen process of the participants. CVD, cardiovascular disease; HFmrEF, heart failure with mildly reduced ejection fraction.

The data of 418 patients aged ≥ 18 years old with HFmrEF were collected. In total, six patients with right cardiac decompensation‐related diseases or primary pulmonary hypertension, 14 patients with valvular heart disease or receiving atrioventricular valve replacement, two patients with congenital heart disease, eight patients receiving mechanical circulation or ventilation support and hemodialysis, two patients complicated with tumor, six patients with severe infection, and one patient with poor quality of ultrasound image were excluded. Finally, 379 patients were included. The screening process of the participants is exhibited in Figure 2.

The mean age in patients with the VMT score of 3 was older than patients with the VMT score of 2 group and the VMT score <2 group (74.13 years vs. 69.70 years vs. 66.11 years). The percentages of patients in different NYHA classifications were statistically different among the VMT score of 3, the VMT score of 2 group, and the VMT score <2 group. The percentage of patients with MR (75.93% vs. 72.73% vs. 28.57%) or TR (68.52% vs. 50.45% vs. 27.62%) in the VMT score of 3 group was higher than the VMT score of 2 group and the VMT score <2 group. The mean E/e' in the VMT score of 3 group was higher than the VMT score of 2 group and the VMT score <2 group (18.08% vs. 16.86% vs. 10.29%). More detailed information of patients was shown in Table 1.

**Table 1:** Comparisons of characteristics in HFmrEF patients with and without all‐cause mortality as well as CVD‐cause mortality.

Variables	Total (*n* = 379)	VMT < 2 (*n* = 105)	VMT of 2 (*n* = 220)	VMT of 3 (*n* = 54)	Statistics	*p*
**Demographic variables**						
Age, years, mean (± SD)	69.34 (± 13.57)	66.11 (± 13.46)	69.70 (± 13.34)	74.13 (± 13.34)	*F* = 6.594	.002
Sex, *n* (%)					*χ*² = 5.678	.058
Female	143 (37.73%)	31 (29.52%)	86 (39.09%)	26 (48.15%)		
Male	236 (62.27%)	74 (70.48%)	134 (60.91%)	28 (51.85%)		
Height, cm, mean (± SD)	165.16 (± 9.10)	165.77 (± 8.61)	165.65 (± 8.88)	161.98 (± 10.38)	*H* = 6.654	.036
Weight, kg, mean (± SD)	69.37 (± 15.11)	68.88 (± 14.99)	71.25 (± 14.73)	62.63 (± 15.17)	*F* = 7.379	.001
BMI, kg/m^2^, *n* (%)					*χ*² = 17.810	.001
BMI ≤ 23.9	153 (40.37%)	44 (41.9%)	74 (33.64%)	35 (64.81%)		
23.9 < BMI ≤ 27.9	136 (35.88%)	38 (36.19%)	87 (39.55%)	11 (20.37%)		
BMI > 27.9	90 (23.75%)	23 (21.9%)	59 (26.82%)	8 (14.81%)		
Smoking, *n* (%)					*χ*² = 0.460	.795
No	170 (44.85%)	50 (47.62%)	96 (43.64%)	24 (44.44%)		
Yes	209 (55.15%)	55 (52.38%)	124 (56.36%)	30 (55.56%)		
Drinking, *n* (%)					*χ*² = 4.352	.114
No	283 (74.67%)	84 (80.00%)	164 (74.55%)	35 (64.81%)		
Yes	96 (25.33%)	21 (20.00%)	56 (25.45%)	19 (35.19%)		
**Complications**						
Hypertension, *n* (%)					*χ*² = 3.024	.220
No	102 (26.91%)	33 (31.43%)	59 (26.82%)	10 (18.52%)		
Yes	277 (73.09%)	72 (68.57%)	161 (73.18%)	44 (81.48%)		
Diabetes, *n* (%)					*χ*² = 0.688	.709
No	196 (51.72%)	56 (53.33%)	110 (50.00%)	30 (55.56%)		
Yes	183 (48.28%)	49 (46.67%)	110 (50.00%)	24 (44.44%)		
Ischemic heart disease, *n* (%)					*χ*² = 16.470	<.001
No	60 (15.83%)	4 (3.81%)	47 (21.36%)	9 (16.67%)		
Yes	319 (84.17%)	101 (96.19%)	173 (78.64%)	45 (83.33%)		
Primary myocardiopathy, *n* (%)					‐	.148
No	365 (96.31%)	104 (99.05%)	210 (95.45%)	51 (94.44%)		
Yes	14 (3.69%)	1 (0.95%)	10 (4.55%)	3 (5.56%)		
Hyperlipemia, *n* (%)					*χ*² = 7.788	.020
No	91 (24.01%)	24 (22.86%)	46 (20.91%)	21 (38.89%)		
Yes	288 (75.99%)	81 (77.14%)	174 (79.09%)	33 (61.11%)		
Peripheral vascular disease, *n* (%)					*χ*² = 0.461	.794
No	241 (63.59%)	65 (61.90%)	143 (65.00%)	33 (61.11%)		
Yes	138 (36.41%)	40 (38.10%)	77 (35.00%)	21 (38.89%)		
Stroke, *n* (%)					*χ*² = 9.266	.010
No	295 (77.84%)	92 (87.62%)	166 (75.45%)	37 (68.52%)		
Yes	84 (22.16%)	13 (12.38%)	54 (24.55%)	17 (31.48%)		
AFIB, *n* (%)					*χ*² = 42.549	<.001
No	260 (68.60%)	96 (91.43%)	140 (63.64%)	24 (44.44%)		
Yes	119 (31.40%)	9 (8.57%)	80 (36.36%)	30 (55.56%)		
History of heart failure, *n* (%)					*χ*² = 29.254	<.001
No	193 (50.92%)	77 (73.33%)	94 (42.73%)	22 (40.74%)		
Yes	186 (49.08%)	28 (26.67%)	126 (57.27%)	32 (59.26%)		
**Clinical data**						
NYHA classification, *n* (%)					*H* = 71.032	<.001
2	158 (41.69%)	79 (75.24%)	70 (31.82%)	9 (16.67%)		
3	94 (24.80%)	16 (15.24%)	63 (28.64%)	15 (27.78%)		
4	127 (33.51%)	10 (9.52%)	87 (39.55%)	30 (55.56%)		
Clinical symptoms of heart failure, *n* (%)					*χ*² = 12.449	.002
Hypoperfusion	84 (22.16%)	28 (26.67%)	36 (16.36%)	20 (37.04%)		
Congestion	295 (77.84%)	77 (73.33%)	184 (83.64%)	34 (62.96%)		
**Laboratory data**						
CRP, mg/L, mean (± SD)	24.58 (± 35.03)	19.80 (± 31.19)	22.10 (± 31.64)	43.97 (± 47.49)	*H* = 15.100	.001
LAC, mmol/L, M (Q₁, Q₃)	1.20 (1.00–1.80)	1.10 (0.90–1.80)	1.2 (1–1.72)	1.7 (1.2–2.65)	*H* = 20.847	<.001
Hemoglobin, g/L, mean (± SD)	122.20 (± 24.92)	128.40 (± 19.91)	120.93 (± 25.40)	115.30 (± 29.18)	*H* = 9.587	.008
Red blood cell, 10^12^/L, mean (± SD)	4.05 (± 0.76)	4.25 (± 0.62)	4.01 (± 0.77)	3.81 (± 0.90)	*H* = 9.562	.008
White blood cell, 10^9^/L, mean (± SD)	8.39 (± 3.53)	8.59 (± 3.32)	8.12 (± 3.30)	9.11 (± 4.60)	*H* = 1.663	.435
Platelet, 10^9^/L, mean (± SD)	209.61 (± 75.98)	224.20 (± 77.47)	211.32 (± 76.45)	174.26 (± 59.47)	*F* = 8.132	<.001
Neutrophil, 10^9^/L, mean (± SD)	6.12 (± 3.20)	6.21 (± 3.14)	5.84 (± 3.00)	7.03 (± 3.89)	*H* = 3.785	.151
Lymphocyte, 10^9^/L, mean (± SD)	1.50 (± 0.66)	1.61 (± 0.56)	1.50 (± 0.67)	1.29 (± 0.76)	*H* = 13.944	.001
Monocyte, 10^9^/L, mean (± SD)	0.59 (± 0.31)	0.62 (± 0.33)	0.56 (± 0.28)	0.60 (± 0.38)	*F* = 1.310	.271
Eosinophil, 10^9^/L, mean (± SD)	0.11 (± 0.12)	0.11 (± 0.10)	0.13 (± 0.13)	0.07 (± 0.10)	*H* = 16.249	<.001
Basophil, 10^9^/L, mean (± SD)	0.02 (± 0.01)	0.02 (± 0.01)	0.02 (± 0.02)	0.02 (± 0.01)	*F* = 1.090	.337
Hematocrit, %, mean (± SD)	36.47 (± 6.95)	38.33 (± 5.44)	36.14 (± 7.11)	34.19 (± 8.07)	*H* = 11.545	.003
BUN, mmol/L, mean (± SD)	8.98 (± 5.96)	6.97 (± 4.33)	9.07 (± 5.48)	12.55 (± 8.43)	*H* = 36.348	<.001
Creatinine, μmol/L, M (Q₁, Q₃)	96.00 (77.80–137.80)	86.40 (71.30–102.30)	67.37 (± 32.78)	47.16 (± 26.05)	*H* = 50.910	<.001
eGFR, ml/min, mean (± SD)	69.33 (± 33.55)	84.85 (± 31.25)	97.95 (78.2–140.7)	141.00 (110.47–181.75)	*H* = 48.491	<.001
Potassium, mmol/L, mean (± SD)	4.13 (± 0.52)	4.03 (± 0.37)	4.18 (± 0.56)	4.08 (± 0.58)	*H* = 4.351	.114
Sodium, mmol/L, mean (± SD)	138.82 (± 3.73)	138.98 (± 3.28)	138.90 (± 3.86)	138.23 (± 4.01)	*F* = 0.805	.448
Calcium, mmol/L, M (Q₁, Q₃)	2.20 (2.11–2.28)	2.24 (2.15–2.32)	2.20 (2.10–2.26)	2.17 (2.10–2.20)	*H* = 19.648	<.001
CK‐MB, U/L, M (Q₁, Q₃)	18.80 (12.60–56.55)	24.00 (13.80–94.40)	16.3 (12–41.88)	22.2 (13.32–40.73)	*H* = 12.559	.002
MB, ng/mL, mean (± SD)	211.69 (± 258.62)	168.13 (± 205.40)	212.16 (± 276.60)	294.46 (± 259.51)	*H* = 16.901	<.001
CTNI, ng/mL, mean (± SD)	11.43 (± 21.21)	14.56 (± 21.86)	8.88 (± 18.44)	15.71 (± 28.29)	*H* = 17.517	<.001
NT‐proBNP, pg/mL, mean (± SD)	7373.88 (± 9492.28)	3526.76 (± 5488.67)	6789.18 (± 8334.44)	17236.47 (± 12908.78)	*H* = 74.648	<.001
**Echocardiographic data**						
MR, *n* (%)					*χ*² = 64.159	<.001
No	148 (39.05%)	75 (71.43%)	60 (27.27%)	13 (24.07%)		
Yes	231 (60.95%)	30 (28.57%)	160 (72.73%)	41 (75.93%)		
TR, *n* (%)					*χ*² = 26.932	<.001
No	202 (53.3%)	76 (72.38%)	109 (49.55%)	17 (31.48%)		
Yes	177 (46.7%)	29 (27.62%)	111 (50.45%)	37 (68.52%)		
LVEDV, mL, M (Q₁, Q₃)	136.00 (110.00–167.00)	122.00 (104.00–143.00)	144 (117–176)	135 (118.75–165)	*H* = 22.429	<.001
LVEF, %, mean (± SD)	45.21 (± 2.47)	45.24 (± 2.33)	45.32 (± 2.48)	44.66 (± 2.65)	*F* = 1.604	.203
IVS, mm, mean (± SD)	10.79 (± 2.24)	10.46 (± 2.32)	11.02 (± 2.18)	10.51 (± 2.21)	*F* = 2.750	.065
LVMI, g/m^2^, mean (± SD)	125.96 (± 35.00)	111.41 (± 29.68)	130.15 (± 35.26)	137.19 (± 35.35)	*F* = 14.386	<.001
SV, mL, M (Q₁, Q₃)	64.70 (52.05–80.30)	58.20 (50.20–67.10)	69.60 (56.67–81.15)	66.15 (51.2–85.6)	*H* = 20.924	<.001
Transmitral peak E, cm/s, mean (± SD)	99.21 (± 29.68)	68.51 (± 16.90)	109.13 (± 25.50)	118.50 (± 19.48)	*H* = 164.264	<.001
EDT, ms, mean (± SD)	142.41 (± 51.64)	136.63 (± 55.68)	144.71 (± 52.15)	144.30 (± 40.06)	*H* = 2.308	.315
TRPG, mmHg, mean (± SD)	31.93 (± 13.06)	24.17 (± 6.86)	33.12 (± 13.74)	42.17 (± 10.60)	*H* = 80.286	<.001
LAVI, mL/m^2^, mean (± SD)	47.14 (± 21.80)	32.94 (± 10.63)	49.80 (± 18.06)	63.97 (± 33.22)	*H* = 104.009	<.001
E/e', mean (± SD)	15.21 (± 4.49)	10.29 (± 2.12)	16.86 (± 3.75)	18.08 (± 3.04)	*H* = 203.748	<.001
Diameter of right ventricular base, cm, mean (± SD)	32.80 (± 5.03)	29.74 (± 3.29)	33.69 (± 4.90)	35.10 (± 5.72)	*H* = 66.479	<.001
TAPSE, mm, mean (± SD)	19.28 (± 3.48)	20.15 (± 2.75)	19.54 (± 3.38)	16.52 (± 3.84)	*H* = 32.100	<.001
RVFAC, %, mean (± SD)	45.07 (± 8.08)	48.02 (± 7.50)	45.39 (± 7.62)	38.02 (± 6.82)	*F* = 32.319	<.001
IVC respiratory change, %, mean (± SD)	60.74 (± 14.99)	70.05 (± 5.94)	62.05 (± 13.69)	37.31 (± 4.71)	*H* = 138.359	<.001
**Medication during hospitalization**						
Intravenous diuretics, *n* (%)					‐	<.001
No	33 (8.71%)	26 (24.76%)	7 (3.18%)	0 (0%)		
Yes	346 (91.29%)	79 (75.24%)	213 (96.82%)	54 (100%)		
Inotropic agents, *n* (%)					*χ*² = 32.812	<.001
No	293 (77.31%)	102 (97.14%)	152 (69.09%)	39 (72.22%)		
Yes	86 (22.69%)	3 (2.86%)	68 (30.91%)	15 (27.78%)		
**Medication after discharge**						
Beta‐blocker, *n* (%)					*χ*² = 23.642	<.001
No	78 (20.58%)	13 (12.38%)	41 (18.64%)	24 (44.44%)		
Yes	301 (79.42%)	92 (87.62%)	179 (81.36%)	30 (55.56%)		
ACEI, *n* (%)					*χ*² = 17.519	<.001
No	321 (84.7%)	76 (72.38%)	195 (88.64%)	50 (92.59%)		
Yes	58 (15.3%)	29 (27.62%)	25 (11.36%)	4 (7.41%)		
ARB, *n* (%)					*χ*² = 6.748	.034
No	277 (73.09%)	80 (76.19%)	151 (68.64%)	46 (85.19%)		
Yes	102 (26.91%)	25 (23.81%)	69 (31.36%)	8 (14.81%)		
**Outcomes**						
Overall survival, *n* (%)					*χ*² = 58.864	<.001
Yes	311 (82.06%)	99 (31.83%)	187 (85.00%)	25 (46.30%)		
No	68 (17.94%)	6 (8.82%)	33 (15.00%)	29 (53.70%)		
CVD survival, *n* (%)					*χ*² = 44.069	<.001
Yes	336 (88.65%)	102 (30.36%)	200 (90.91%)	34 (62.96%)		
No	43 (11.35%)	3 (6.98%)	20 (9.09%)	20 (37.04%)		

Abbreviations: ACEI, angiotensin converting enzyme inhibitor; AFIB, atrial fibrillation; ARB, angiotensin receptor blockers; BMI, body mass index; BUN, blood urea nitrogen; CK‐MB, creatine kinase isoenzyme; CRP, C‐reactive protein; CTNI, cardiac troponin I; CVD, cardiovascular disease; EDT, deceleration time of E; eGFR, estimate glomerular filtration rate; HFmrEF, heart failure with mildly reduced ejection fraction; IHD, ischemic heart disease; IVS, interventricular septum; K, kalium; LAC, lactic acid; LAVI, left atrial volume index; LVEDV, left ventricular end‐diastolic volume; LVEF, left ventricular ejection fraction; LVMI, left ventricular mass index; M, median; MB, myoglobin; MR, mitral regurgitation; Na, sodium; NT‐proBNP, amino‐terminal pro‐brain natriuretic peptide; NYHA, New York Heart Association; Q₁, 1st quartile; Q₃, 3st quartile; PCM, primary cardiac myopathy; PVD, peripheral vascular diseases; RVFAC, right ventricular fractional area change; SD, standard deviation; SV, stroke volume; *t*, Student's *t* test; *t*’, Satterthwaite *t* test; TAPSE, tricuspid annular plane systolic excursion; TR, tricuspid regurgitation; TRPG, tricuspid regurgitation pressure gradient; VMT, mitral valve and tricuspid valve opening; W, Wilcoxon rank sum test; *χ*², chi‐square test; ‐, Fisher's exact test.

### 3.2 The association between the VMT score and 1‐year all‐cause mortality in patients with HFmrEF

As presented in Supporting Information S5: Table 1, age, gender, ischemic heart disease, hyperlipemia, AFIB, NYHA classification, clinical symptoms of HF, CRP, LAC, hemoglobin, red blood cell, platelet, neutrophil, lymphocyte, monocyte, eosinophil, basophil, BUN, eGFR, sodium, calcium, MB, CTNI, amino‐terminal NT‐proBNP, MR, TR, early‐diastolic velocity (E), TRPG, LVAI, E/e', TAPSE, RVFAC, IVC respiratory change, inotropic agents, beta‐blocker, ACEI, and ARB were potential covariates correlated with all‐cause mortality in patients with HFmrEF.

The results of stepwise regression revealed that age, clinical symptoms, platelet, neutrophil, lymphocyte, hematocrit, NT‐proBNP, LAVI, and inotropic agents were confounding factors for all‐cause mortality in patients with HFmrEF. In the crude model, the VMT score of 2 might be a risk factor for all‐cause mortality in patients with HFmrEF (HR = 2.74, 95%CI: 1.15–6.54). The VMT score of 3 might increase the risk of all‐cause mortality in patients with HFmrEF (HR = 12.29, 95%CI: 5.09–29.65). After adjusting for confounding factors, elevated risk of all‐cause mortality in HFmrEF patients was identified in those with the VMT score of 2 (HR = 3.01, 95%CI: 1.13–7.99), and the VMT score of 3 (HR = 5.02, 95%CI: 1.89–13.32). In model 3, AFIB was further adjusted based on model 2, the risk of all‐cause mortality was increased in HFmrEF patients in the VMT score of 2 group (HR = 2.80, 95%CI: 1.04–7.52) and the VMT score of 3 group (HR = 4.29, 95%CI: 1.58–11.66) (Table 2). As exhibited in Supporting Information S1: Figure 1, the survival probability of patients with a VMT score < 2 was higher than those with a VMT score of 2 and a VMT score of 3. The AUC of the VMT score for predicting 1‐year all‐cause mortality of HFmrEF patients was 0.724 (95%CI: 0.663–0.785) (Supporting Information S2: Figure 2).

**Table 2:** The association between the VMT score and 1‐year all‐cause mortality in patients with HFmrEF.

Variables	Model 1		Model 2		Model 3	
	HR (95% CI)	*p*	HR (95% CI)	*p*	HR (95% CI)	*p*
VMT score						
VMT < 2	Ref		Ref		Ref	
VMT of 2	2.74 (1.15–6.54)	.023	3.01 (1.13–7.99)	.027	2.80 (1.04–7.52)	.041
VMT of 3	12.29 (5.09–29.65)	<.001	5.02 (1.89–13.32)	.001	4.29 (1.58–11.66)	.004

*Note*: Model 1: the unadjusted model; Model 2: adjusting for age, clinical symptoms, platelet, neutrophil, lymphocyte, hematocrit, NT‐proBNP, LAVI, and inotropic agents; and Model 3: adjusting for age, clinical symptoms, platelet, neutrophil, lymphocyte, hematocrit, NT‐proBNP, LAVI, AFIB, and inotropic agents.

Abbreviations: AFIB, atrial fibrillation; CI, confidence interval; HFmrEF, heart failure with mildly reduced ejection fraction; HR, hazard ratio; LAVI, left atrial volume index; NT‐proBNP, amino‐terminal pro‐brain natriuretic peptide; VMT, mitral valve, and tricuspid valve opening.

### 3.3 The association between the VMT score and 1‐year CVD‐cause mortality in patients with HFmrEF

Age, hyperlipemia, HYHA classification, clinical symptoms, CRP, LAC, neutrophil, lymphocyte, monocyte, eosinophil, basophil, BUN, eGFR, sodium, calcium, MB, NT‐proBNP, MR, TR, LVEF, transmitral peak E, TRPG, LAVI, E/e', TAPSE, RVFAC, IVC respiratory change, and beta‐blocker were potential confounders for 1‐year CVD‐cause mortality in patients with HFmrEF (Supporting Information S5: Table 2).

After stepwise regression, the covariates associated with 1‐year CVD‐cause mortality in patients with HFmrEF were age, neutrophil, eosinophil, BUN, eGFR, CNTI, and LVEF. In the unadjusted model, the VMT score of 3 might be a risk factor for 1‐year CVD‐cause mortality in patients with HFmrEF (HR = 17.05, 95%CI: 5.05–57.49). The increased risk of 1‐year CVD‐cause mortality in patients with HFmrEF was found in patients with the VMT score of 3 after the adjustment of covariates (HR = 6.51, 95%CI: 1.50–28.22). We further adjusted AFIB based on model 2, and found that the VMT score of 3 were associated with an increased risk of 1‐year CVD‐cause mortality in patients with HFmrEF (HR = 7.63, 95%CI: 1.70–34.33) (Table 3). The Kaplan–Meier curve depicted that the survival probability of patients in the VMT score of 3 group was lower than those in the VMT score of 2 and the VMT score <2 group (Supporting Information S3: Figure 3). The AUC of the VMT score for predicting 1‐year CVD‐cause mortality of HFmrEF patients was 0.748 (95%CI: 0.676–0.819) (Supporting Information S4: Figure 4).

**Table 3:** The association between the VMT score and 1‐year CVD‐cause mortality in patients with HFmrEF.

Variables	Model 1		Model 2		Model 3	
	HR (95% CI)	*p*	HR (95% CI)	*p*	HR (95% CI)	*p*
VMT score						
VMT < 2	Ref		Ref		Ref	
VMT of 2	3.33 (0.99–11.20)	.052	3.26 (0.84–12.62)	0.087	3.16 (0.82–12.23)	.096
VMT of 3	17.05 (5.05–57.49)	<.001	6.51 (1.50–28.22)	0.012	7.63 (1.70–34.33)	.008

*Note*: Model 1: the unadjusted model; Model 2: adjusting for age, neutrophil, eosinophil, BUN, eGFR, CNTI, and LVEF and Model 3: adjusting for age, neutrophil, eosinophil, BUN, eGFR, CNTI, AFIB, and LVEF.

Abbreviations: AFIB, atrial fibrillation; BUN, blood urea nitrogen; CI, confidence interval; CTNI, cardiac troponin I; CVD, cardiovascular disease; eGFR, estimate glomerular filtration rate; HFmrEF, heart failure with mildly reduced ejection fraction; HR, hazard ratio; LVEF, left ventricular ejection fraction; VMT, mitral valve, and tricuspid valve opening.

## 4 DISCUSSION

In the current study, the associations between the VMT score and 1‐year all‐cause or CVD‐cause mortality of HFmrEF patients were assessed. The predictive values of the VMT score for the 1‐year all‐cause and CVD‐cause mortality in HFmrEF patients were also evaluated. The results showed that elevated risk of all‐cause mortality in HFmrEF patients was identified in those with a VMT score ≥2, and increased CVD‐cause mortality in HFmrEF patients was identified in those with a VMT score of 3. The survival probability of patients with a VMT score <2 was higher than those with a VMT score of 2 and a VMT score of 3. The AUC of the VMT score for predicting 1‐year all‐cause mortality of HFmrEF patients was 0.724, and for predicting 1‐year CVD‐cause mortality of HFmrEF patients was 0.748. The findings might provide a quick tool to evaluate the prognosis of patients with HFmrEF, and for those with a high risk of mortality, relevant interventions should be timely provided.

There was evidence revealing that signs of elevated LVFP, such as the presence of lung congestion, invasively measured pulmonary artery wedge pressure, and echocardiographic diastolic function assessed in a stable state, were prognostic markers for hospitalized HF patients irrespective of EF.^16^, ^17^ Previously, several echocardiographic parameters were recognized to be markers of LVFP, and echocardiography was an essential method for the evaluation of LVFP and management of patients with HF.^18^ Nevertheless, the diagnosis of elevated LVFP in HF patients is still challenging.^19^ While the algorithm recommended by the current guidelines has been invasively validated in large multicenter studies both in HFrEF and HFpEF,^20^ and whether this algorithm was suitable for HFmrEF patients was unknown.^21^

In normal circumstances, the early diastolic opening of the TV precedes that of the MV, but as a major factor for HF, once LVFP is elevated, MV opening happens early and precedes TV opening, resulting from an early crossover of LA and LV pressures.^22^, ^23^ The increase of LVFP can also lead to secondary postcapillary pulmonary hypertension, resulting in increased right ventricular pressure, impaired right ventricular diastolic function, and prolonged right ventricular diastolic time.^24^ The time delay of RV inflow relative to the LV inflow, as evaluated by the dual‐Doppler system, was found to be an index of LVFP in patients with HF.^22^ The correlation between the change of MV‐TV opening time interval obtained from ultrasound examination and LVFP was identified based on the hemodynamic information, suggesting the simple algorithm of VMT scoring could be used as a novel parameter of LVFP.^12^ When the VMT score was ≥2, MV opening happens earlier than TV opening, or MT and TV simultaneous opening, accompanied by abnormal RA pressure, which was consistent with hemodynamic changes caused by LVFP increase. Moreover, the VMT score ≥2 was confirmed to be correlated with the prognosis of HFpEF population^13^ These gave evidence to the results in our study, which revealed that the VMT score ≥2 was associated with elevated risk of 1‐year all‐cause and the VMT score of 3 was correlated with increased risk of CVD‐cause mortality. The VMT score has good predictive value for all‐cause and CVD‐cause mortality in HFmrEF patients. For HFmrEF patients with atrial fibrillation who cannot evaluate LVFP using conventional algorithms, the VMT score might help evaluate the LVFP status. Compared with the application value of two‐dimensional ultrasound indicators such as E/A, E/e', and isovolumic relaxation time in HF,^15^, ^25^ the VMT score is more closely related to the pathophysiological mechanism and hemodynamic changes of HF, which can avoid the limitations of pseudo‐normalization of conventional indicators in evaluating LVFP. Thus, the VMT score might provide another choice of diagnostic methods for patients with HFmEF.

Due to the mildly reduced ejection fraction and the increase in residual volume after ventricular ejection in patients with HFmrEF, the left ventricular end‐systolic volume (LVESV) increases, and ventricular filling is limited, while the increase of LVFP might further aggravate the increase of LVESV and accelerate the process of cardiac remodeling. Due to the heterogeneity of clinical manifestations in HFmrEF patients, the assessment of their prognosis is relatively difficult, and there is no effective method recommended by guidelines. In our study, the VMT score ≥2 was identified as a prognostic marker for the prognosis of HFmrEF, which was expected to add a diagnostic option for HFmrEF patients. The VMT score was easy to calculate according to echocardiography, and the AUC value of the VMT score for predicting 1‐year all‐cause mortality of HFmrEF patients was 0.724, and for predicting 1‐year CVD‐cause mortality of HFmrEF patients was 0.748. This study confirms that the application of the VMT score can help rapidly and accurately evaluate the LVFP elevation in HFmrEF patients, which is helpful for the early identification of high‐risk patients with poor prognoses. The VMT score can provide clinical guidance for early intervention in HFmrEF patients with a high risk of poor prognosis, standardize the comprehensive management of HF, and help delay the process of ventricular remodeling in patients to improve poor prognosis.

There were several limitations in this study. Firstly, this was a single‐center retrospective study with a small sample size, which might cause selection bias. Secondly, the results of the VMT score depended on the resolution of the two‐dimensional echocardiogram, and high spatial resolution images were required. Thirdly, the temporal resolution of images was required as the VMT score was based on timing of atrioventricular valves. The results should be interpreted with caution and if necessary, higher resolution might be required.

## 5 CONCLUSIONS

The current study evaluated the prognostic value of the VMT score for 1‐year all‐cause or CVD‐cause mortality of HFmrEF patients. The results showed that HFmrEF patients with a VMT score <2 had a higher survival probability of patients than those with a VMT score ≥2. The VMT score presented good predictive value for 1‐year all‐cause or CVD‐cause mortality of HFmrEF patients.

## CONFLICT OF INTEREST STATEMENT

The authors declare no conflict of interest.

## Supporting information

Supplementary Figure 1 The Kaplan‐Meier curve showing the 1‐year survival probability of HFmrEF patients in different VMT score group.

Supplementary Figure 2 The ROC of VMT for 1‐year all‐cause mortality of HFmrEF patients.

Supplementary Figure 3 The Kaplan‐Meier curve of the CVD‐cause mortality of HFmrEF patients in different VMT score group.

Supplementary Figure 4 The ROC of VMT for CVD‐cause mortality of HFmrEF patients.

Supporting information.

## Data Availability

The data that support the findings of this study are available from the corresponding author upon reasonable request.
