# Supplementary figures and images for: The prognostic value of the visually assessed time difference between mitral valve and tricuspid valve opening score for patients with heart failure with mildly reduced ejection fraction
Source: Clin Cardiol. 2024 Jan 29;47(2):10.1002/clc.24223. doi: 10.1002/clc.24223 (PMC10823457; doi:10.1002/clc.24223)

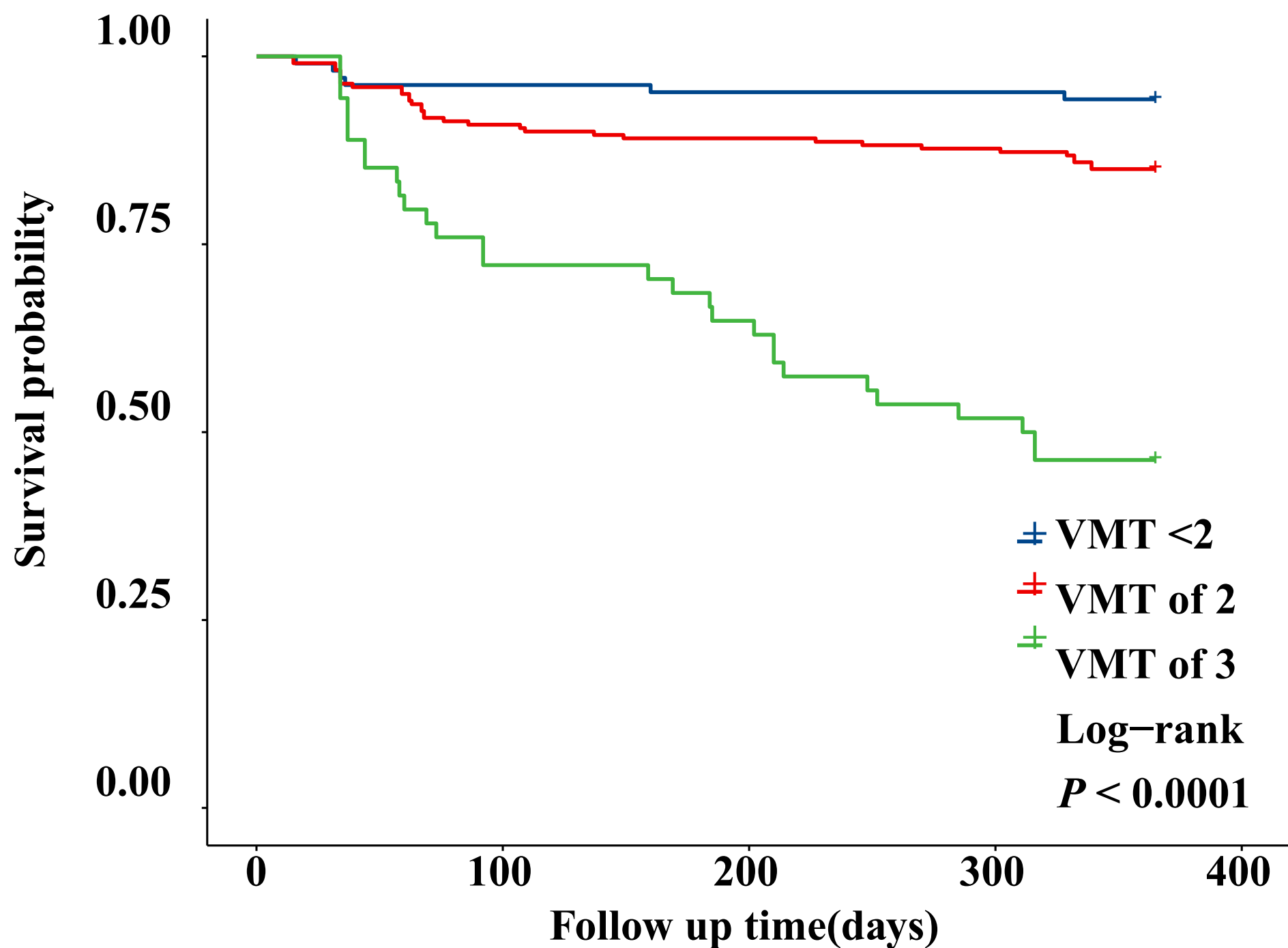

**Number at risk: n (%)**

|                  |                             |          |          |          |       |
|------------------|-----------------------------|----------|----------|----------|-------|
| <b>VMT &lt;2</b> | 105 (100)                   | 101 (96) | 100 (95) | 100 (95) | 0 (0) |
| <b>VMT of 2</b>  | 220 (100)                   | 200 (91) | 196 (89) | 193 (88) | 0 (0) |
| <b>VMT of 3</b>  | 54 (100)                    | 39 (72)  | 35 (65)  | 28 (52)  | 0 (0) |
|                  | 0                           | 100      | 200      | 300      | 400   |
|                  | <b>Follow up time(days)</b> |          |          |          |       |

Supplement: Supplementary file 1 — Supplementary Figure 1 The Kaplan‐Meier curve showing the 1‐year survival probability of HFmrEF patients in different VMT score group. [file CLC-47--s002.pdf]

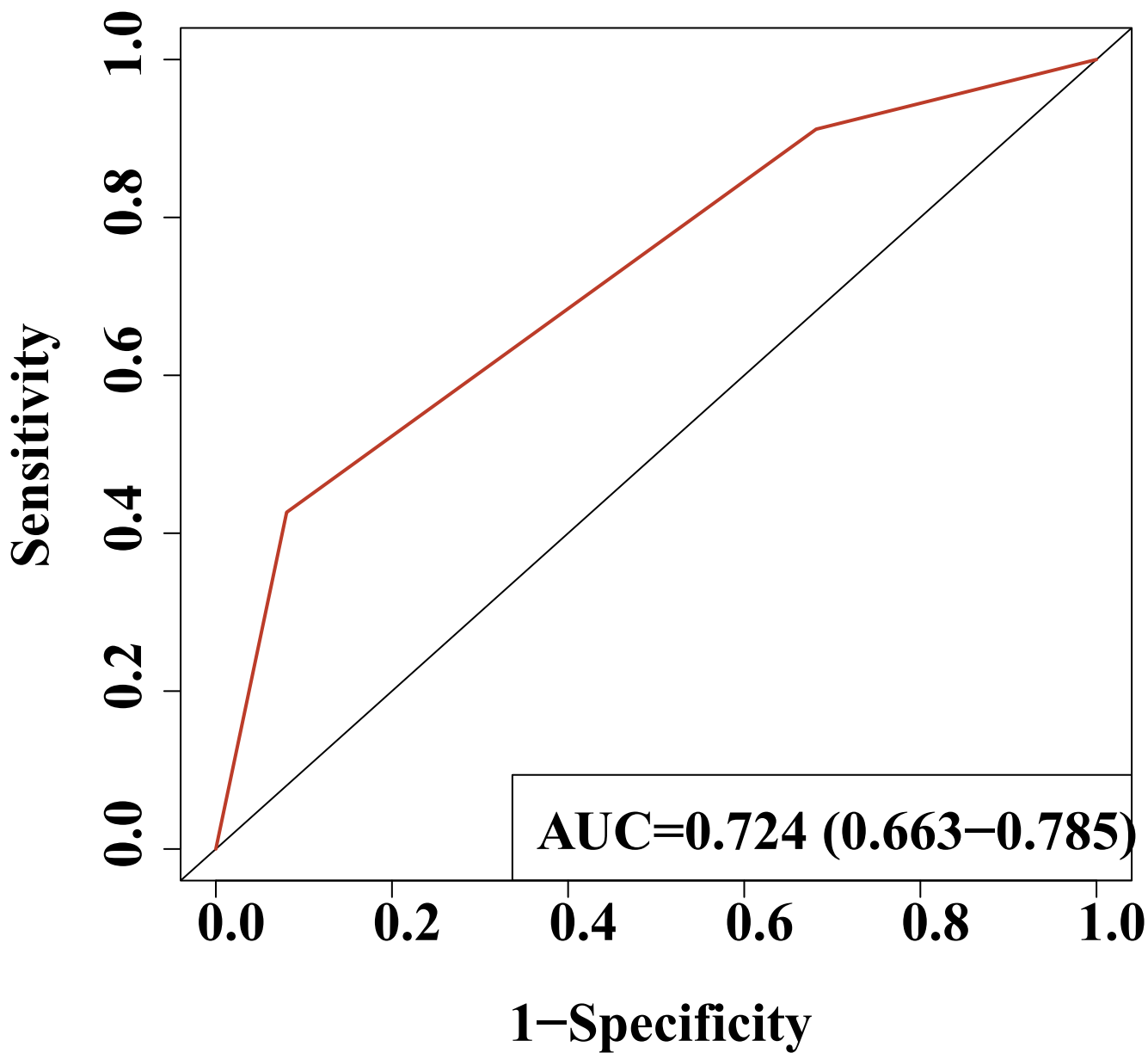

Supplement: Supplementary file 2 — Supplementary Figure 2 The ROC of VMT for 1‐year all‐cause mortality of HFmrEF patients. [file CLC-47--s004.pdf]

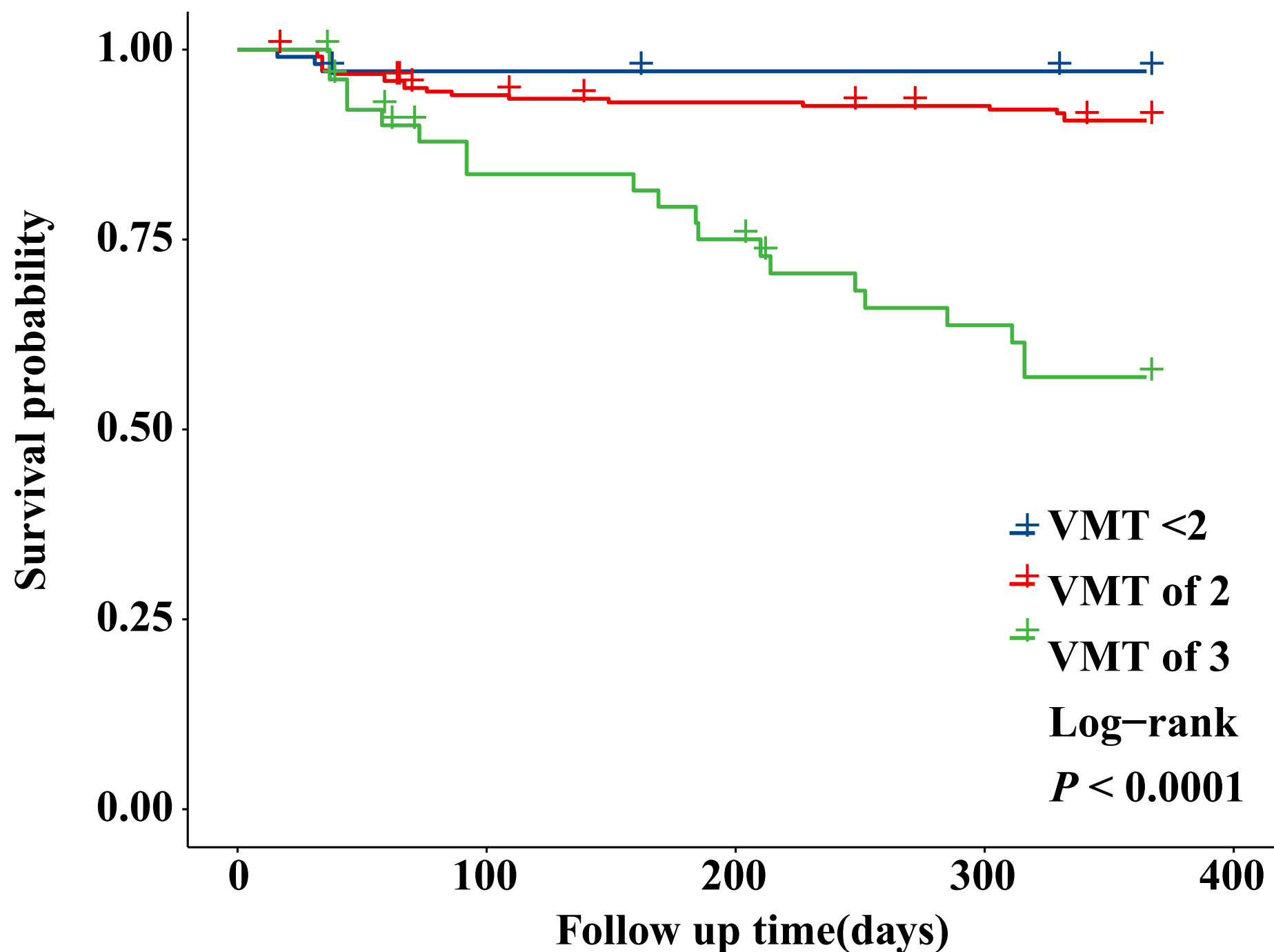

| Number at risk: n (%) |           |          |          |          |       |
|-----------------------|-----------|----------|----------|----------|-------|
|                       | 0         | 100      | 200      | 300      | 400   |
| <b>VMT &lt;2</b>      | 105 (100) | 101 (96) | 100 (95) | 100 (95) | 0 (0) |
| <b>VMT of 2</b>       | 220 (100) | 200 (91) | 196 (89) | 193 (88) | 0 (0) |
| <b>VMT of 3</b>       | 54 (100)  | 39 (72)  | 35 (65)  | 28 (52)  | 0 (0) |
|                       | 0         | 100      | 200      | 300      | 400   |
| Follow up time(days)  |           |          |          |          |       |

Supplement: Supplementary file 3 — Supplementary Figure 3 The Kaplan‐Meier curve of the CVD‐cause mortality of HFmrEF patients in different VMT score group. [file CLC-47--s005.pdf]

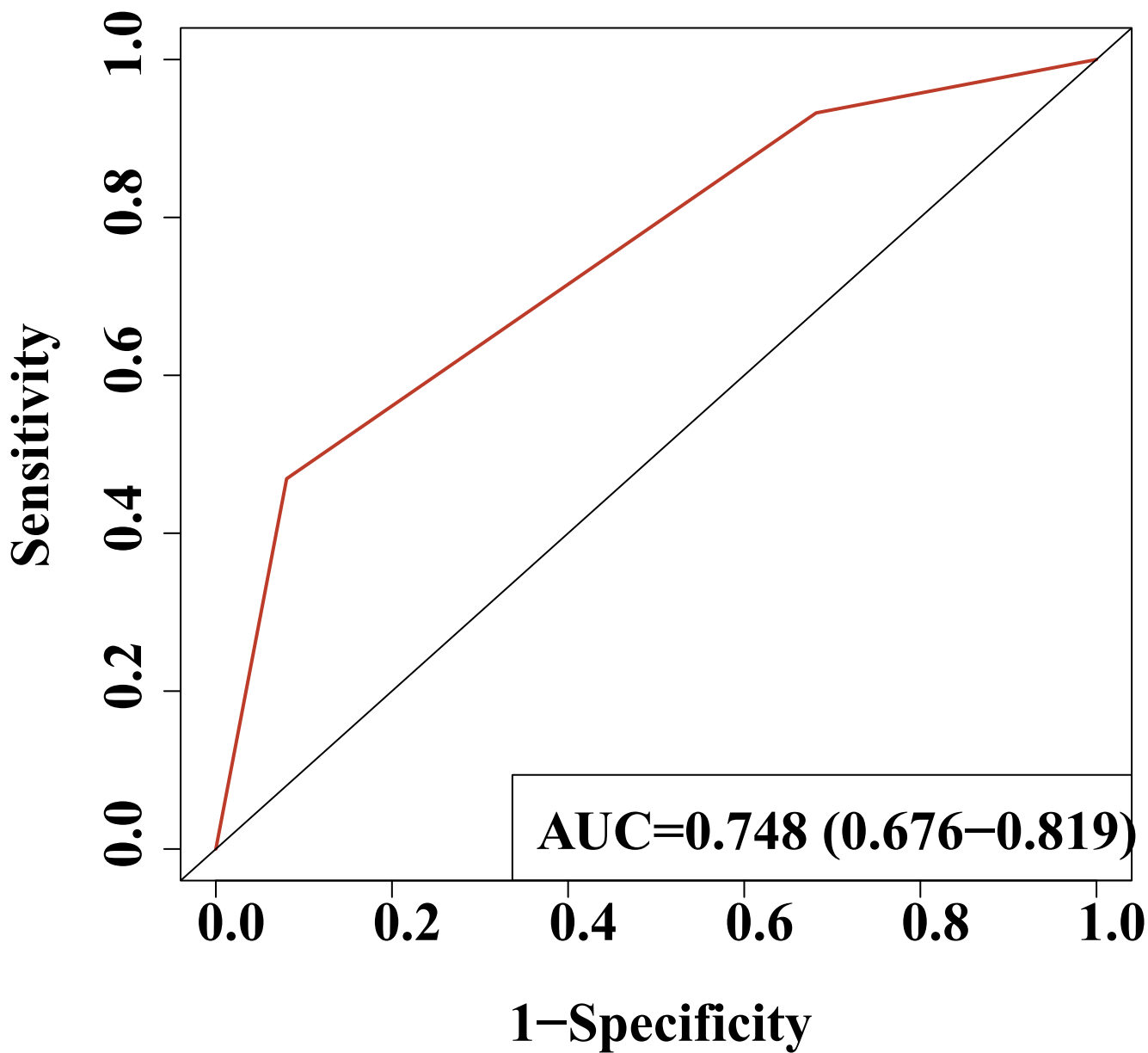

Supplement: Supplementary file 4 — Supplementary Figure 4 The ROC of VMT for CVD‐cause mortality of HFmrEF patients. [file CLC-47--s001.pdf]
