# Supplementary material for: The prognostic value of the visually assessed time difference between mitral valve and tricuspid valve opening score for patients with heart failure with mildly reduced ejection fraction
Source: Clin Cardiol. 2024 Jan 29;47(2):10.1002/clc.24223. doi: 10.1002/clc.24223 (PMC10823457; doi:10.1002/clc.24223)
Supplement: Supplementary file 5 — Supporting information. [file CLC-47--s003.docx]

Supplementary Table 1 Potential confounding factors associated with all-cause mortality

| Variables | HR (95% CI) | *P* |
| --- | --- | --- |
| Age | 3.11 (2.17-4.46) | <0.001 |
| Sex |  |  |
| Female | Ref |  |
| Male | 0.53 (0.33-0.86) | 0.010 |
| BMI |  |  |
| BMI≤23.9 | Ref |  |
| 23.9-27.9 | 0.95 (0.56-1.59) | 0.837 |
| BMI>27.9 | 0.57 (0.29-1.13) | 0.108 |
| Smoking |  |  |
| No | Ref |  |
| Yes | 1.08 (0.67-1.75) | 0.749 |
| Drinking |  |  |
| No | Ref |  |
| Yes | 1.41 (0.85-2.35) | 0.181 |
| Hypertension |  |  |
| No | Ref |  |
| Yes | 1.22 (0.70-2.14) | 0.486 |
| Diabetes |  |  |
| No | Ref |  |
| Yes | 0.74 (0.46-1.20) | 0.217 |
| IHD |  |  |
| No | Ref |  |
| Yes | 3.30 (1.20-9.06) | 0.021 |
| PCM |  |  |
| No | Ref |  |
| Yes | 1.15 (0.36-3.67) | 0.810 |
| Hyperlipemia |  |  |
| No | Ref |  |
| Yes | 0.55 (0.33-0.90) | 0.018 |
| PVD |  |  |
| No | Ref |  |
| Yes | 1.14 (0.70-1.86) | 0.588 |
| Stroke |  |  |
| No | Ref |  |
| Yes | 1.16 (0.67-2.01) | 0.591 |
| AFIB |  |  |
| No | Ref |  |
| Yes | 2.41 (1.49-3.87) | <0.001 |
| History of heart failure |  |  |
| No | Ref |  |
| Yes | 0.76 (0.47-1.23) | 0.261 |
| NYHA |  |  |
| 2 | Ref |  |
| 3 | 1.85 (0.95-3.60) | 0.068 |
| 4 | 2.66 (1.48-4.78) | 0.001 |
| Clinical symptoms |  |  |
| Hypoperfusion | Ref |  |
| Congestion | 0.28 (0.18-0.46) | <0.001 |
| CRP | 1.57 (1.33-1.85) | <0.001 |
| LAC | 1.53 (1.36-1.72) | <0.001 |
| Hemoglobin | 0.53 (0.42-0.66) | <0.001 |
| Red blood cell | 0.52 (0.41-0.66) | <0.001 |
| Platelet | 0.74 (0.56-0.98) | 0.033 |
| Neutrophil | 1.76 (1.47-2.11) | <0.001 |
| Lymphocyte | 0.45 (0.32-0.62) | <0.001 |
| Monocyte | 1.29 (1.06-1.57) | 0.010 |
| Eosinophil | 0.24 (0.14-0.41) | <0.001 |
| Basophil | 0.42 (0.28-0.63) | <0.001 |
| Hematocrit | 0.50 (0.40-0.63) | <0.001 |
| BUN | 1.56 (1.35-1.79) | <0.001 |
| eGFR | 0.49 (0.37-0.65) | <0.001 |
| Potassium | 1.21 (0.97-1.51) | 0.099 |
| Sodium | 0.58 (0.46-0.74) | <0.001 |
| Calcium | 0.77 (0.66-0.90) | 0.001 |
| CK-MB | 1.07 (0.90-1.27) | 0.456 |
| MB | 1.70 (1.47-1.96) | <0.001 |
| CTNI | 1.52 (1.30-1.78) | <0.001 |
| NT-proBNP | 1.79 (1.56-2.06) | <0.001 |
| MR |  |  |
| No | Ref |  |
| Yes | 4.76 (2.36-9.60) | <0.001 |
| TR |  |  |
| No | Ref |  |
| Yes | 2.10 (1.29-3.44) | 0.003 |
| LVEDV | 0.84 (0.63-1.11) | 0.219 |
| LVEF | 1.08 (0.85-1.38) | 0.541 |
| IVS | 0.84 (0.66-1.07) | 0.153 |
| LVMI | 1.06 (0.84-1.34) | 0.636 |
| SV | 0.89 (0.69-1.17) | 0.412 |
| E peak | 1.49 (1.17-1.88) | 0.001 |
| EDT | 1.00 (0.79-1.27) | 0.982 |
| TRPG | 1.51 (1.25-1.83) | <0.001 |
| LAVI | 1.46 (1.24-1.73) | <0.001 |
| E/e’ | 1.45 (1.17-1.81) | 0.001 |
| Diameter of right ventricular base | 1.03 (0.82-1.31) | 0.775 |
| TAPSE | 0.69 (0.56-0.87) | 0.001 |
| RVFAC | 0.72 (0.56-0.92) | 0.008 |
| IVC respiratory change | 0.48 (0.39-0.60) | <0.001 |
| Intravenous diuretics |  |  |
| No | Ref |  |
| Yes | 3.39 (0.83-13.83) | 0.089 |
| Inotropic agents |  |  |
| No | Ref |  |
| Yes | 0.43 (0.20-0.89) | 0.023 |
| Beta-blocker |  |  |
| No | Ref |  |
| Yes | 0.27 (0.16-0.43) | <0.001 |
| ACEI |  |  |
| No | Ref |  |
| Yes | 0.16 (0.04-0.63) | 0.009 |
| ARB |  |  |
| No | Ref |  |
| Yes | 0.48 (0.25-0.91) | 0.026 |

HR: Hazard ratio; CI: Confidence intervals; Ref: reference; BMI: Body mass index; IHD: Ischemic heart disease; PCM: Primary cardiac myopathy; PVD: Peripheral vascular diseases; AFIB: atrial fibrillation; NYHA: New York Heart Association; CRP: C-reactive protein; LAC: Lactic acid; BUN: Blood urea nitrogen; eGFR: Estimate glomerular filtration rate; K: Kalium; Na: Sodium; CK-MB: Creatine kinase Isoenzyme; MB: Myoglobin; CTNI: Cardiac troponin I; NT-proBNP: amino-terminal pro-brain natriuretic peptide; MR: Mitral regurgitation; TR: Tricuspid regurgitation; LVEDV: Left ventricular end-diastolic volume; LVEF: Left ventricular ejection fraction; IVS: Interventricular septum; LVMI: Left ventricular mass index; SV: Stroke volume; EDT: Deceleration time of E; TRPG: Tricuspid regurgitation pressure gradient; LAVI: Left atrial volume index; TAPSE: Tricuspid annular plane systolic excursion; RVFAC: Right ventricular fractional area change; ACEI: Angiotensin converting enzyme inhibitor; ARB: Angiotensin receptor blockers

Supplementary Table 2 Potential confounding factors associated with CVD-cause mortality

| Variables | HR (95% CI) | *P* |
| --- | --- | --- |
| Age | 4.27 (2.56-7.12) | <0.001 |
| Sex |  |  |
| Female | Ref |  |
| Male | 0.71 (0.39-1.31) | 0.274 |
| BMI |  |  |
| BMI≤23.9 | Ref |  |
| 23.9-27.9 | 0.90 (0.47-1.74) | 0.755 |
| BMI>27.9 | 0.56 (0.24-1.33) | 0.188 |
| Smoking |  |  |
| No | Ref |  |
| Yes | 1.23 (0.67-2.27) | 0.501 |
| Drinking |  |  |
| No | Ref |  |
| Yes | 1.76 (0.95-3.26) | 0.074 |
| Hypertension |  |  |
| No | Ref |  |
| Yes | 0.70 (0.37-1.31) | 0.265 |
| Diabetes |  |  |
| No | Ref |  |
| Yes | 0.56 (0.30-1.06) | 0.074 |
| IHD |  |  |
| No | Ref |  |
| Yes | 2.75 (0.85-8.88) | 0.091 |
| PCM |  |  |
| No | Ref |  |
| Yes | 1.88 (0.58-6.07) | 0.292 |
| Hyperlipemia |  |  |
| No | Ref |  |
| Yes | 0.50 (0.27-0.93) | 0.030 |
| PVD |  |  |
| No | Ref |  |
| Yes | 0.84 (0.44-1.59) | 0.587 |
| Stroke |  |  |
| No | Ref |  |
| Yes | 1.20 (0.60-2.38) | 0.605 |
| AFIB |  |  |
| No | Ref |  |
| Yes | 2.41 (1.49-3.87) | <0.001 |
| History of heart failure |  |  |
| No | Ref |  |
| Yes | 0.76 (0.42-1.39) | 0.375 |
| NYHA |  |  |
| 2 | Ref |  |
| 3 | 1.93 (0.82-4.54) | 0.133 |
| 4 | 3.02 (1.43-6.37) | 0.004 |
| Clinical symptoms |  |  |
| Hypoperfusion | Ref |  |
| Congestion | 0.19 (0.10-0.34) | <0.001 |
| CRP | 1.45 (1.17-1.81) | 0.001 |
| LAC | 1.54 (1.33-1.78) | <0.001 |
| Hemoglobin | 0.85 (0.62-1.16) | 0.304 |
| Red blood cell | 0.81 (0.59-1.10) | 0.172 |
| Platelet | 0.86 (0.62-1.20) | 0.377 |
| Neutrophil | 1.99 (1.61-2.47) | <0.001 |
| Lymphocyte | 0.68 (0.47-0.97) | 0.034 |
| Monocyte | 1.49 (1.21-1.84) | <0.001 |
| Eosinophil | 0.04 (0.01-0.13) | <0.001 |
| Basophil | 0.43 (0.26-0.71) | 0.001 |
| Hematocrit | 0.81 (0.60-1.10) | 0.182 |
| BUN | 1.49 (1.23-1.79) | <0.001 |
| eGFR | 0.53 (0.38-0.75) | <0.001 |
| Potassium | 1.22 (0.93-1.61) | 0.156 |
| Sodium | 0.50 (0.37-0.67) | <0.001 |
| Calcium | 0.80 (0.64-0.99) | 0.038 |
| CK-MB | 1.10 (0.91-1.33) | 0.338 |
| MB | 1.77 (1.48-2.10) | <0.001 |
| CTNI | 1.67 (1.40-2.00) | <0.001 |
| NT-proBNP | 1.83 (1.54-2.18) | <0.001 |
| MR |  |  |
| No | Ref |  |
| Yes | 3.18 (1.48-6.86) | 0.003 |
| TR |  |  |
| No | Ref |  |
| Yes | 2.07 (1.11-3.84) | 0.021 |
| LVEDV | 0.69 (0.47-1.03) | 0.068 |
| LVEF | 1.48 (1.09-2.01) | 0.013 |
| IVS | 0.84 (0.62-1.14) | 0.270 |
| LVMI | 0.96 (0.71-1.31) | 0.815 |
| SV | 0.77 (0.53-1.11) | 0.158 |
| E peak | 1.50 (1.12-2.02) | 0.007 |
| EDT | 1.17 (0.89-1.53) | 0.275 |
| TRPG | 1.60 (1.27-2.02) | <0.001 |
| LAVI | 1.58 (1.31-1.91) | <0.001 |
| E/e’ | 1.39 (1.05-1.83) | 0.021 |
| Diameter of right ventricular base | 1.05 (0.78-1.40) | 0.765 |
| TAPSE | 0.51 (0.39-0.67) | <0.001 |
| RVFAC | 0.47 (0.35-0.64) | <0.001 |
| IVC respiratory change | 0.46 (0.34-0.61) | <0.001 |
| Intravenous diuretics |  |  |
| No | Ref |  |
| Yes | 4.32 (0.59-31.40) | 0.148 |
| Inotropic agents |  |  |
| No | Ref |  |
| Yes | 0.62 (0.28-1.40) | 0.250 |
| Beta-blocker |  |  |
| No | Ref |  |
| Yes | 0.24 (0.13-0.44) | <0.001 |
| ACEI |  |  |
| No | Ref |  |
| Yes | 0.25 (0.06-1.03) | 0.055 |
| ARB |  |  |
| No | Ref |  |
| Yes | 0.48 (0.21-1.08) | 0.077 |

HR: Hazard ratio; CI: Confidence intervals; Ref: reference; BMI: Body mass index; IHD: Ischemic heart disease; PCM: Primary cardiac myopathy; PVD: Peripheral vascular diseases; AFIB: atrial fibrillation; NYHA: New York Heart Association; CRP: C-reactive protein; LAC: Lactic acid; BUN: Blood urea nitrogen; eGFR: Estimate glomerular filtration rate; K: Kalium; Na: Sodium; CK-MB: Creatine kinase Isoenzyme; MB: Myoglobin; CTNI: Cardiac troponin I; NT-proBNP: amino-terminal pro-brain natriuretic peptide; MR: Mitral regurgitation; TR: Tricuspid regurgitation; LVEDV: Left ventricular end-diastolic volume; LVEF: Left ventricular ejection fraction; IVS: Interventricular septum; LVMI: Left ventricular mass index; SV: Stroke volume; EDT: Deceleration time of E; TRPG: Tricuspid regurgitation pressure gradient; LAVI: Left atrial volume index; TAPSE: Tricuspid annular plane systolic excursion; RVFAC: Right ventricular fractional area change; ACEI: Angiotensin converting enzyme inhibitor; ARB: Angiotensin receptor blockers
